# Decoding multiple myeloma: single-cell insights into tumor heterogeneity, immune dynamics, and disease progression

**DOI:** 10.3389/fimmu.2025.1584350

**Published:** 2025-05-08

**Authors:** Zhenzhen Zhao, Zhijie Zhao, Zhiheng Lin, Lu Fan, Zhikai Xiahou, Yujiang Dong, Weiying Bao

**Affiliations:** ^1^ The First Clinical Medical College of Shandong University of Traditional Chinese Medicine, Jinan, China; ^2^ Department of Plastic and Reconstructive Surgery, Shanghai Ninth People’s Hospital, Shanghai JiaoTong University School of Medicine, Shanghai, China; ^3^ Department of Gynecology, Longhua Hospital, Shanghai University of Traditional Chinese Medicine, Shanghai, China; ^4^ Department of Hematology, Songjiang Hospital Affiliated to Shanghai Jiao Tong University School of Medicine, Shanghai, China; ^5^ China Institute of Sport and Health Science, Beijing Sport University, Beijing, China; ^6^ The Second Affiliated Hospital of Shandong University of Chinese Medicine, Department of Cardiovascular Disease, Jinan, China

**Keywords:** ScRNA-seq, neuroimmunity, NR3C1, IGLC3, APP, MIF, multiple myeloma

## Abstract

**Background:**

Multiple myeloma (MM) is a biologically heterogeneous malignancy of clonal plasma cells, often progressing from MGUS or smoldering MM. It causes anemia, bone lesions, and immune dysfunction due to abnormal plasma cell expansion in the bone marrow. Neuroinflammatory and neurotrophic factors may influence MM progression by affecting immune cells and the bone marrow niche. Growing evidence points to a role for neuroimmune regulation in tumor immunity. Despite therapeutic progress, disease heterogeneity and resistance highlight the need for new strategies targeting the tumor microenvironment and neuroimmune axis.

**Methods:**

This investigation exploited single-cell RNA sequencing (scRNA-seq) to analyze MM and high-risk smoldering multiple myeloma (SMMh) samples, identifying 11 distinct cell types. We examined their transcriptional signatures, stemness, proliferative properties, and metabolic pathways, with particular attention to neuroimmune interactions in the tumor microenvironment. Using trajectory inference tools such as CytoTRACE, Monocle2, and Slingshot, we traced the differentiation paths of MM cell subpopulations and identified key signaling pathways that may influence immune responses and tumor progression.

**Results:**

The analysis identified four distinct subpopulations of myeloma cells, with the C0 *IGLC3+* myeloma cells representing the least differentiated and most proliferative subset. These cells played a critical role in MM progression and may contribute to immune evasion mechanisms. Additionally, receptor-ligand interactions within the tumor microenvironment were identified, which may be influenced by neuroinflammatory and neurotrophic factors. These findings suggest that the nervous system and immune modulation significantly affect tumor biology, highlighting potential therapeutic targets that could be exploited to overcome resistance to conventional therapies.

**Conclusion:**

This single-cell analysis provided new insights into the cellular diversity and differentiation trajectories in MM, offering a deeper understanding of the complex neuroimmune interactions that drive tumor progression and resistance. By incorporating the role of neuroinflammation and immune modulation, our study suggested novel therapeutic strategies targeting the neuroimmune axis in oncology, ultimately contributing to the development of more effective, personalized treatment approaches for MM.

## Introduction

Cancer neuroimmunology is an emerging discipline that investigates the intricate relationships involving the central nervous system and cancer immunity. Recent research has transformed the perspective on the nervous system, not just as a bystander, instead as a crucial regulator of malignancy and therapeutic responses (1). The core characteristics of the immune microenvironment of MM, such as myeloid-derived suppressor cells (MDSCs) accumulation, T cell exhaustion state, and IL-6 driven immunosuppression. Both the central and peripheral nervous systems contribute to modulating the tumor microenvironment, influencing neuroinflammation, immune responses, and immune evasion mechanisms (2). Interactions in hematological tumors, such as multiple myeloma (MM), are significant, as the immune system’s microsetting is critical to disease progression and therapeutic resistance.

MM is a kind of plasma cell carcinoma that develops mostly in its bone marrow, which accounts for roughly ten percent of all hematologic malignancies (3, 4). MM precursor lesions include monoclonal gammopathy, which is of unknown significance, as well as smoldering multiple myeloma (SMMh) (5). Of these, SMM can progress to symptomatic MM. MM is diagnosed with a ≥10% proportion of monoclonal plasma cells in the bone marrow or a biopsy-confirmed plasmacytoma with end-organ damage (6).

The distinguishing feature of MM is the aberrant proliferation of monoclonal plasma cells in the bone marrow, resulting in the creation of large numbers of nonfunctional immunoglobulins or their light chains (7). These abnormal plasma cells trigger clinical manifestations such as anemia, bone lesions, infections, hypercalcemia, and renal failure by interacting with other cells in the bone marrow microcosm (8). Despite enormous advances in the medical management and prognosis of MM in the past decade, its bioheterogeneity and drug resistance mechanisms remain a barrier for research (9, 10).

The neurological system has a vital function in regulating the immune response to MM. Neuroimmune interactions occur through neurotransmitters and neuropeptides, which can influence immune cell function and tumor progression. For example, neuroinflammation, induced by factors such as stress, can alter immune responses, promoting tumor growth and resistance to therapies. Furthermore, the nervous system can impact immune evasion mechanisms in MM, such as through immune checkpoint molecules such as PD-L1, which are expressed upon the cells of the MM, which inhibits T-cell activity (9, 11). These emerging insights highlight the neuroimmune axis as a critical area for exploring new therapeutic strategies in MM.

MM treatment includes chemotherapy, targeted therapy, immunotherapy, bone marrow transplantation, and supportive care (12). Immunotherapy is particularly important in MM, and the main strategies include: immune checkpoint inhibition, in which MM cells evade immune killing by inhibiting T-cell activity through the expression of PD-L1 (13). Cytokine modulation, by secreting cytokines such as IL-6, MM cells inhibit immune cell function and promote self-proliferation (14). Innovative immunotherapies, such as CAR-T cell therapy targeting BCMA and bispecific immunoglobulins, albeit showing great success, must still overcome difficulties, including resistance to medicines and negative reactions (14).

The heterogeneity of MM stems from complex interactions between cellular subpopulations within the tumor and the bone marrow microenvironment, and traditional bulk sequencing methods struggle to capture differences at the single-cell level (15). Single-cell RNA sequencing (16, 17) (scRNA-seq) provides a revolutionary technique for resolving MM, revealing: transcriptional patterns of discrete cellular subpopulations clearly described at the single-cell level (18). Analyze intercellular signaling pathways and key receptor-ligand pairs (19). Identify specific subpopulations with active proliferation, high stemness and significant metabolic features. Importantly, scRNA-seq also provides an opportunity to explore the neuroimmune axis in MM by revealing how neuroinflammatory pathways and immune modulation influence tumor progression and treatment resistance (20–22).

In this study, we used scRNA-seq to examine the cellular heterogeneity of MM, focusing on transcriptional signatures, stemness characteristics, proliferative capacity, and metabolic profiles of MM cells. Using trajectory inference tools such as CytoTRACE, Monocle2, and Slingshot, we mapped the differentiation pathways of MM subpopulations and identified key proliferative and functional subpopulations.

In addition, we investigated the interactions between MM cells and the bone marrow environmental conditions, focusing on neuroimmune interactions and receptor-ligand combinations that may contribute to immune regulation. By uncovering these cellular characteristics and their interplay with neuroinflammation, we aim to provide new insights for precision medicine and the development of novel therapeutic strategies, particularly in the context of neuroimmune modulation (23).

## Materials and methods

### Data sources

During this investigation, we used scRNA-seq data from MM as well as SMMh from the GEO database (GSE124310). Ethical approval numbers were not required for this study as the data were obtained from publicly available databases (24). Open data access helped to avoid the ethical review process.

### Single-cell sequencing

Gene expression data were processed using Seurat in R (25, 26). Poor-quality cells were excluded using specific standards, selecting cells with nFeature counts between 300 and 5000, nCount between 500 and 50,000, and mitochondria or red cell gene expression contributions of less than 10% and 5%, respectively. Following quality control, 13,437 cells remained (27, 28).

### Identification of cell types

The NormalizeData functions of Seurat were used to normalize the data (29–32). FindVariableFeatures was used to identify the 2000 highly variable genes (HVGs) (33–36). The scaleData function standardized the data. Principal component analysis (PCA)was performed on these HVGs using the RunPCA function (37–39), and Harmony package was applied to reduce batch effects. For clustering the reduced data (40–42), the FindNeighbors and FindClusters functions were used. Uniform Manifold Approximation and Projection (UMAP) (43, 44) was used for dimensionality reduction clustering analysis, and the results were displayed in a two-dimensional space. To enhance annotation accuracy, FindAllMarkers was applied alongside reference datasets from the CellMarker database and published literature for single-cell annotation.

### Cell stemness assessment

AUCell was a strategy for finding cells containing genes with activity in scRNA-seq data. The “activity” of each cell’s genes was produced by AUCell using a set of genes as input. of genes as input. The stemness level of cell subpopulations was assessed using the AUCell method.

### Gene ontology and gene set enrichment analysis

GO enrichment analysis was a bioinformatics method used to analyze gene function (45–47). Based on the GO database (48–52), it mapped a set of genes to three levels: Biological Process (BP), Molecular Function (MF) and Cellular Component (CC) (53–56).

Differentially expressed genes (DEGs) were found using the FindAllMarkers tool (min.pct = 0.25, threshold = 0.25). Gene enrichment and analysis were carried out utilizing ClusterProfiler, concentrating on important GO keywords with adjusting p-values <0.05. GO descriptions from the National Center for Biotechnology Information, UniProt, and Gene Ontology databases were used to investigate the functional roles of marker genes. To identify key GO categories, the exact Fisher test was used, with FDR corrections included in p-values.

Pathway enrichment was assessed using GSEA software (32, 57, 58) (http://www.gsea-msigdb.org This method ranks DEGs to identify p-values.) This method ranks DEGs to identify significantly enriched pathways between experimental and control groups (59).

### pySCENIC analysis

pySCENIC was a Python-based tool designed for inferring Gene Regulatory Networks (GRNs) and characterizing cell states in single-cell analysis. First, it constructed GRNs by identifying regulatory interactions between transcription factors (TFs) and their target genes. Connection specific index (CSI) was used to analyze the distribution of TFs in myeloma cells. Finally, the AUCell algorithm evaluated TF activity across different cells, quantifying their regulatory influence. These steps collectively provided a comprehensive understanding of the role and regulatory mechanisms of TFs at the single-cell level.

### Metabolic analysis

We evaluated the activity scores of key metabolic pathways in each cell by the AUCell method based on single-cell transcriptome data. The distribution of pathway scores across cell subtypes was compared to identify significantly different metabolic pathways. Pathway scores were averaged for cells within each subtype, and the overall level of activity of specific metabolic pathways in each subtype was calculated.

### Proposed time-series analysis

CytoTRACE infered cell differentiation status by analyzing single-cell RNA-seq data (60–62). It estimates the “stemness” or developmental potential of cells by measuring their transcriptional heterogeneity and identifies cells that are likely at early or late stages of differentiation.

Monocle2 reconstructs cellular differentiation paths (or pseudotime) by performing dimensionality reduction and ordering cells along a trajectory. It can handle branching trajectories and identify genes that are differentially expressed along the progression, useful for studying dynamic biological processes like cell fate decisions. Slingshot constructs tree-like structures to model branching trajectories and is particularly effective when combined with clustering techniques. Monocle2 and Slingshot are more focused on constructing cell differentiation pathways and are suitable for depicting multi-branched developmental trajectories. These methods comprehensively evaluate the differentiation status and developmental potential of MM cell subsets from different dimensions.

### Communication between cells

With the CellChat application, cell-cell interactions between various cell types were predicted. We examined the patterns of incoming and outgoing signals as well as the strength of each receptor-ligand interaction (63).

### Cell culture

KMS-26 and MM1-S were cell lines commonly used in multiple bone marrow studies. KMS-26 cell lines were cultured in RPMI-1640 (Roswell Park Memorial College 1640 medium) or Dulbecco’s Modified Eagle Medium (Dulbecco’s Modified Eagle medium) supplemented with 10% fetal bovine serum (FBS) and 1% penicillin/streptomycin. The MM1-S cell line was cultured in RPMI-1640 supplemented with 10% fetal bovine serum (FBS) and 1% penicillin/streptomycin; the environment was 37°C, 5% CO_2_. The culture environment was 37°C, 5% CO_2_. Cells were passaged every 2–3 days, depending on the cell growth.

### Cell viability assay

Cell viability assay was a method for assessing cell health, viability and proliferation, which is widely used in cell culture, drug screening, toxicity testing and cancer research (64). The assay could quantitatively assess cell viability and determine the effects of different treatment conditions (e.g. drug action, gene knockdown or environmental changes) on cells.

### qPCR

qPCR (65) was a molecular biology technique used to detect and quantify specific nucleic acid sequences (66). Quantitative analysis of nucleic acids was achieved by monitoring the amplification of DNA in real time during the amplification of the PCR reaction using a fluorescent dye or a probe (67, 68).

### Colony formation assay

Colony formation assay was used to detect the proliferation ability of individual cells and was particularly suitable for the study of clone formation ability of tumor cells. The assay was based on the long-term growth of cells in a culture medium, resulting in the formation of a colony that was visible to the naked eye. The colony formation assay was based on the principle that when a single cell undergoes more than six rounds of proliferation *in vitro*, its progeny collectively formed a distinct cluster of cells, referred to as a colony. By computing the colony formation efficiency, the test cells’ ability to proliferate was ascertained.

### Cell scratch assay

Cell migration was measured using the cell scratch assay. The ability of the cells to migrate was assessed by looking at their capacity to fill a “scratch” on a monolayer cell culture dish.

### The transwell assay

The Transwell assay served to investigate a cell’s capacity for invasion and migration. The capacity of cells to move over the membrane from the upper chamber to the lower chamber was evaluated using a Transwell with holes. Cell invasive capacity can be assessed using a Matrigel-coated membrane.

### EdU staining

During DNA replication, the thymidine analog EdU (5-Ethynyl-2’-deoxyuridine) can be added to freshly produced DNA. A fluorescently tagged Click-iT reaction was used to identify EDU-labeled cells and evaluate their capacity for cell proliferation.

### Statistical analysis

Statistical analyses were performed using R packages (69) to process the database counts. Two-tailed p-values were employed, and values below 0.05 were considered statistically significant. * p<0.05, * * p< 0.01, * * * p<0.001, * * * *<0.0001, ns indicates no statistical difference.

## Results

### Heterogeneity of cells in MM as well as SMMh

**Figure 1:**
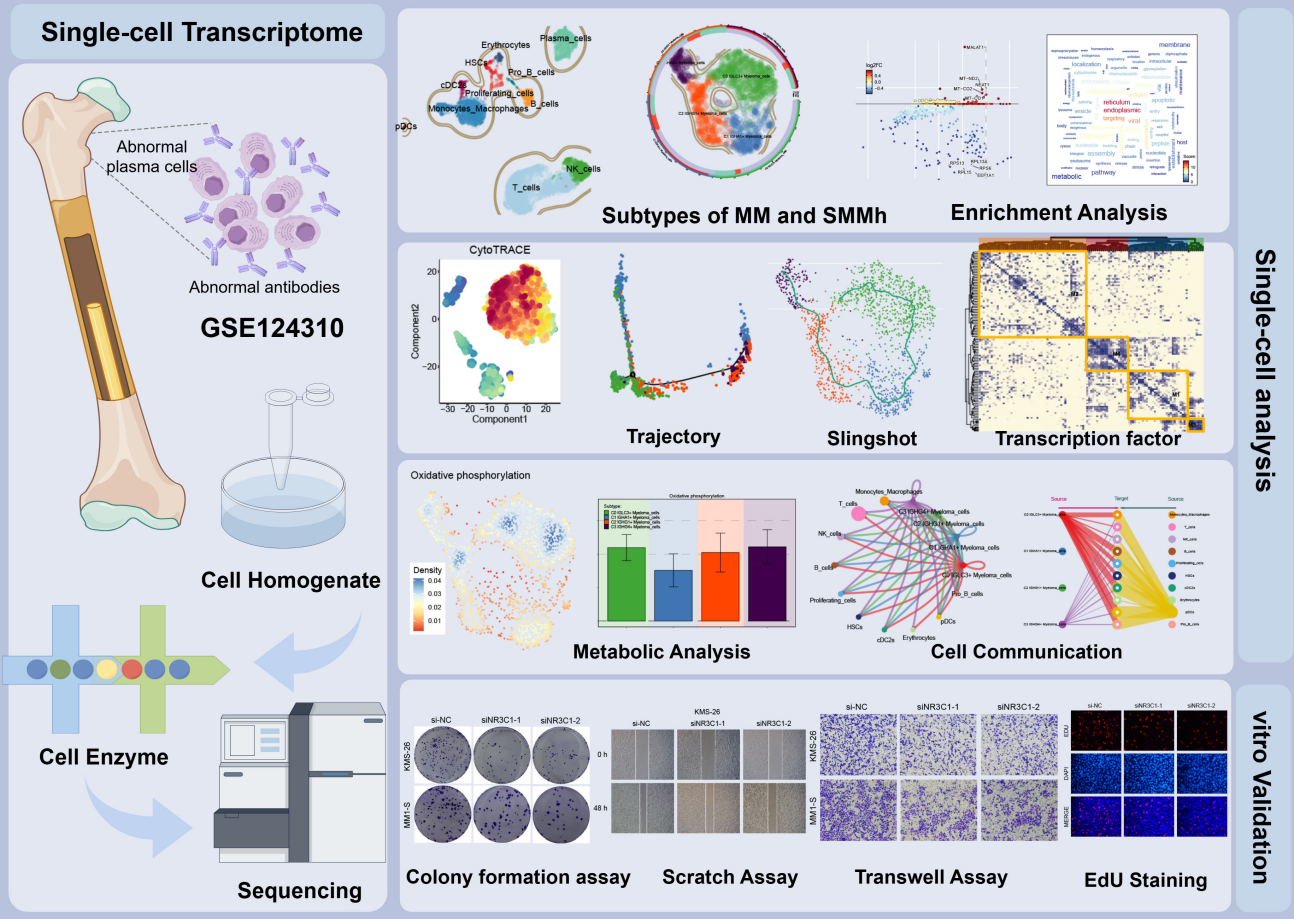
Flow chart of this study. In this study, cellular heterogeneity in MM and SMMh samples was analyzed by single-cell RNA sequencing, identifying 11 cell types. Significant differences in transcriptional profiles were found between MM and SMMh cells, and different cell subpopulations had different regulatory activities in cellular value-addition, differentiation and metabolic pathways. The differentiation process of myeloma cells was speculated using trajectory analysis methods such as CytoTRACE and Monocle2, revealing that C0 *IGLC3+* Myeloma cells are the most naïve tumor cells and that they have a key role in myeloma development and progression. The molecular characterization of myeloma cells during malignant transformation was further elucidated by the analysis of TFs and metabolic pathways.

**Figure 2:**
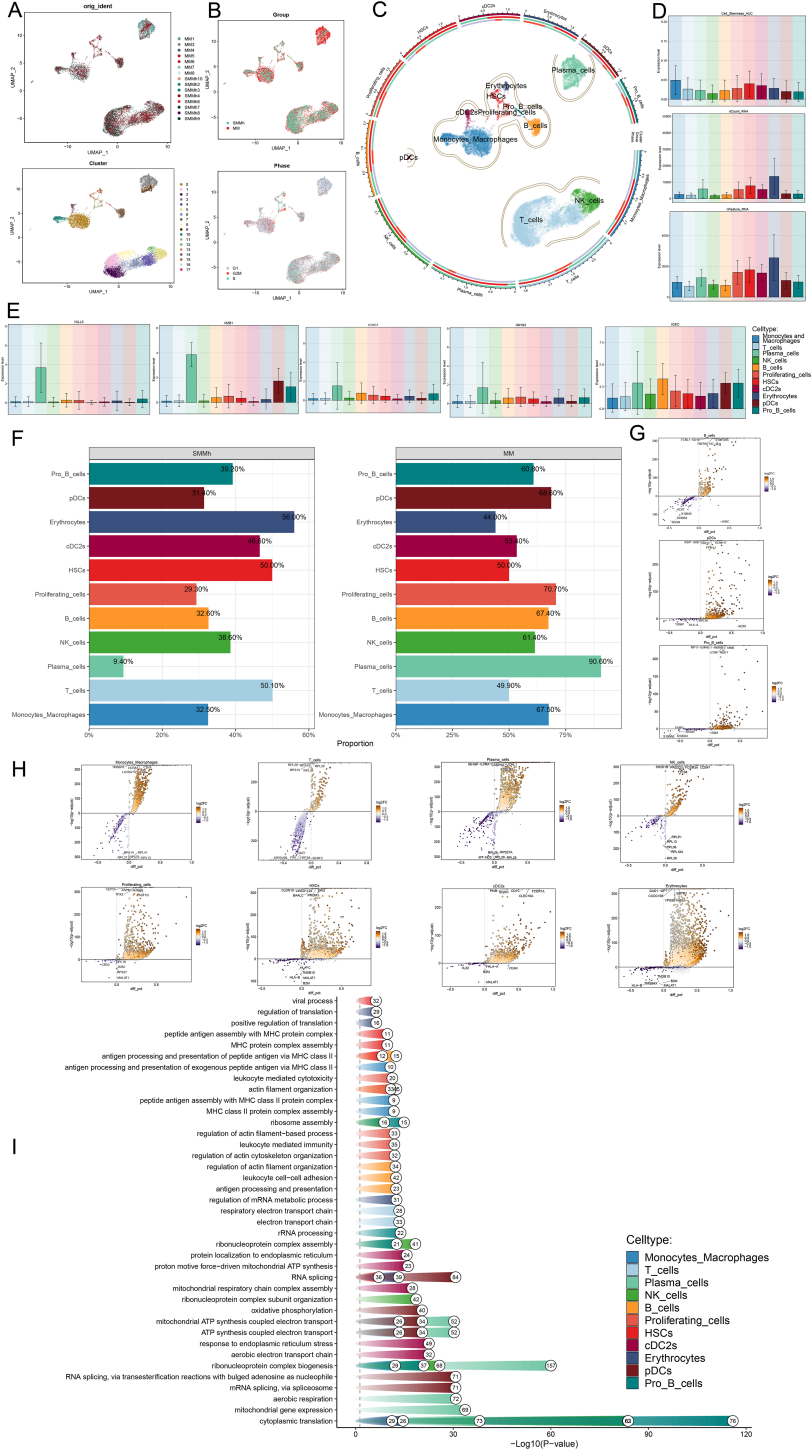
Heterogeneity of cells in MM as well as high-risk smoldering myeloma. **(A)** UMAP plots showed the analysis of all cells from 15 MM and SMMh samples using the scRNA-seq method (upper panel) as well as these samples after dimensionality reduction clustering into 17 cell clusters. **(B)** UMAP plots showed the tissue type of all cells (MM,SMMh) and the cell cycle stage they are in (G1, G2M, S). **(C)** UMAP plot showed 11 different cell types (Monocytes and Macrophages, T cells, Plasma cells, NK cells, B cells, Proliferating cells, HSCs, cDC2s, Erythrocytes, pDCs, Pro B cells). **(D)** Bar plots showed the Cell Stemness AUC (the AUC score of Cell Stemness), nCount RNA, and nFeature RNA scores for 11 cells. **(E)** Bar plots showed the expression of TOP5 marker gene in Plasma cells in all cell types separately. **(F)** Bar plots demonstrated the percentage of SMMh and MM tissue types in each cell type. **(G-H)** Volcano plots demonstrated the expression of up- and down-regulated differential genes in all cell types. **(I)** Enrichment analysis of differential genes in 11 cell types.

First, we showed the overall flow chart of this study (**Figure 1**). By analyzing scRNA-seq data obtained from 15 myeloma patient samples, we successfully identified two tissue types, SMMh cells as well as MM cells. Following rigorous quality control and the removal of batch effects, we were able to extract 13,437 high-quality cells. Then, by further dimensionality reduction clustering analysis, we identified 18 different cell states of these cells (**Figure 2A**). And we labeled the tissue origin (MM,SMMh) as well as the cell cycle order (G1, G2M, S) in which all cells were located (**Figure 2B**). Based on previous studies in the literature and specific biomarkers for each cell type identified by research consensus, we categorized these cell clusters into 11 major cell types: Monocytes and Macrophages (2,556), T cells (6,254), Plasma cells (1696), NK cells (1355), B cells (690), Proliferating cells (222), HSCs (208), cDC2s (178), Erythrocytes (141), pDCs (86), and Pro B cells (51) (**Figure 2C**). Bar plots demonstrated 11 cell types for Cell Stemness AUC (the AUC score of Cell Stemness), nCount RNA, and nFeature RNA scores for 11 cell types. Monocytes and Macrophages, T cells, Plasma cells, HSCs and cDC2s had relatively high Cell Stemness AUC, the levels of nCount RNA and nFeature RNA of Erythrocytes, Proliferating cells, HSCs, cDC2s and Plasma cells were relatively high, which indicates that these cell clusters were active and may be in the stage of cell proliferation and active function (**Figure 2D**). The expression of TOP5 differential genes *IGLL5, MZB1, IGHG1, IGHG3* and *IGKC* in Plasma cells were demonstrated by Bar plots, among which *IGLL5* and *MZB1* were highly expressed in Plasma cells, while IGKC was also highly expressed in other cells (**Figure 2E**). IGLL5 is closely associated with the early stages of B cell development, suggesting a possible role in maintaining the immature state of malignant plasma cells. MZB1 is an endoplasmic reticulum chaperone involved in immunoglobulin assembly, which may be related to secretion load and stress response of MM cells. IGHG1 and IGHG4 represent immunoglobulin heavy chain type changes, reflecting the differences in the differentiation lineages of MM clones. It was worth noting that 90.60% of Plasma cells are from MM (**Figure 2F**). In order to further study the expression of up-regulated and down-regulated genes in all cell types, we showed them by volcano diagrams (**Figures 2G, H**). The up-regulated genes in Plasma cells were *IGHGP, IL5RA, CADPS2, UCHL1* and *KDELR3*, and the down-regulated genes were *RPL28, RPS27A, MT-ND3, RPL39* and *RPL26*. Based on the DEGs, we performed enrichment analysis of related biological processes in different cells. The ATP production coupled with electron mobility, the biogenesis of the ribonucleoprotein complex, mitochondrial gene expression, and other pathways were enriched in plasma cells, while the regulation of actin filament-based operations, leukocyte-mediated immunity, and the organization of the actin cytoskeleton were enriched in proliferating cells (**Figure 2I**). These findings suggest that plasma cells are primarily engaged in energy production and mitochondrial function, whereas proliferating cells are more involved in cytoskeletal organization and immune regulation.

### Single cell characteristics of myeloma cell subsets

**Figure 3:**
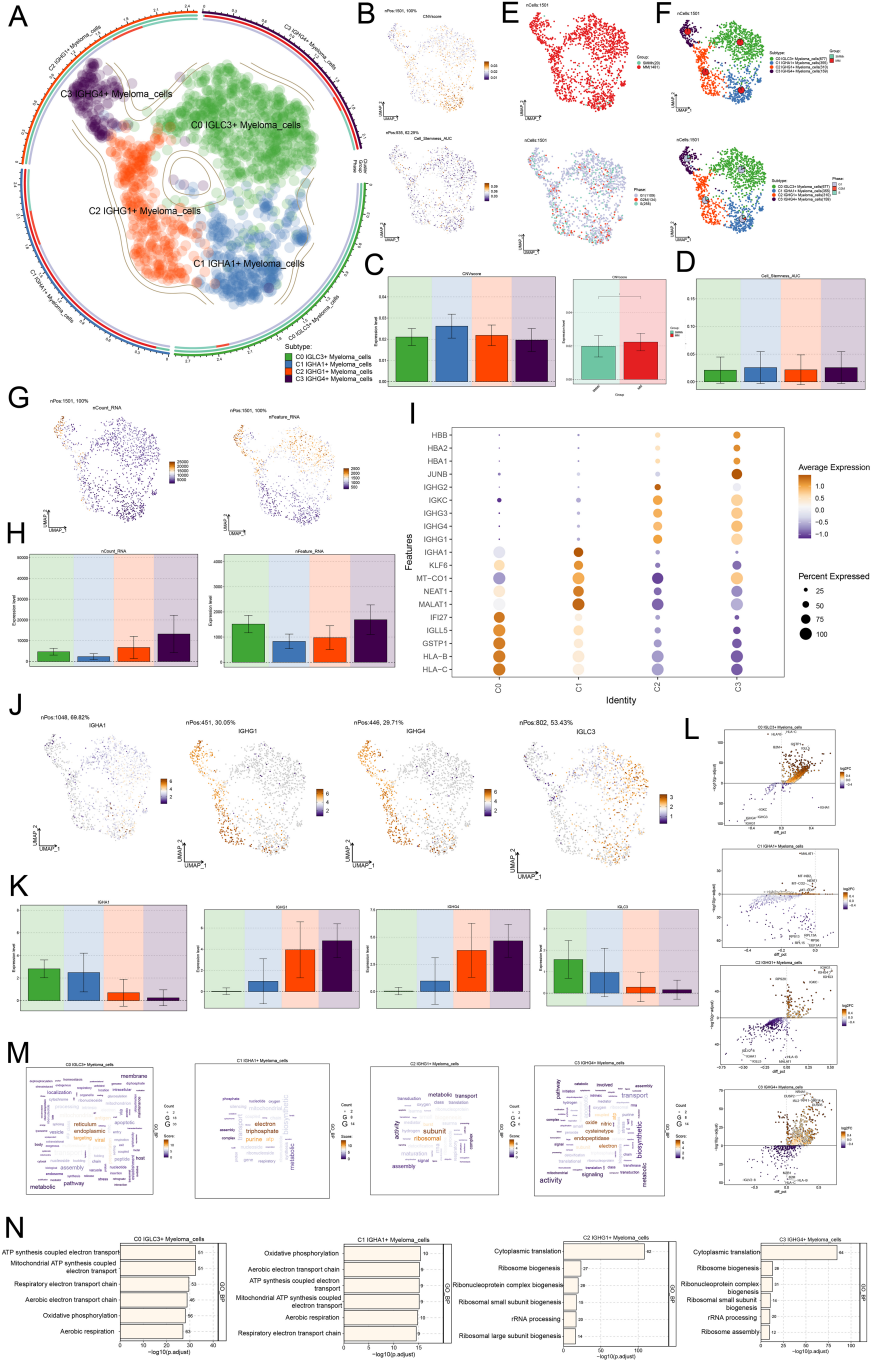
Single cell characteristics of myeloma cell subsets. **(A)** UMAP plot showed the distribution of four myeloma cell subsets. **(B)** UMAP plots showed the difference of CNVscore and Cell Stemness AUC scores of all myeloma cells. **(C)** The bar plots showed the difference of CNVscore of four myeloma cell subsets and different tissue types. **(D)** The bar plot showed the difference of Cell Stemness AUC scores among four myeloma cell subsets. **(E)** The tissue types (SMMh, MM) and the distribution of cell cycle stages (G1, G2M, S) of all myeloma cells. **(F)** UMAP plots showed the distribution of four myeloma cells and pie chart shows the tissue type and the proportion of cell cycle stages of each myeloma cell subpopulation. **(G, H)** UMAP plots and Bar plots showed the differences of nCount RNA and nFeature RNA scores of all myeloma cells. **(I)** Bubble plot showed the expression of TOP5marker gene in four myeloma cell subsets. **(J, K)** UMAP and bar plots showed the expression of *IGLC3, IGHA1, IGHG1, IGHG4* in all myeloma cells. **(L)** Volcano plots showed up-regulated and down-regulated differential genes in four myeloma cell subsets. **(M)** The word cloud plots showed the results of path enrichment. **(N)** The bar plots showed the enrichment analysis results of GO-BP of myeloma cell subsets.

We classified 1501 plasma cells in MM into four distinct cell subsets, C1 *IGHA1+* Myeloma cells, C2 *IGHG1+* Myeloma cells, and C3 *IGHG4+* Myeloma cells, in order to better understand the characteristics of plasma cells in MM (**Figure 3A**). Next, we analyzed the RNA copy number variability of myeloma cell subpopulations as well as the active levels of stemness genes, and found that the differences in CNVscore and Cell Stemness AUC scores among the four myeloma cell subpopulations were small (**Figures 3B–D**). When examining the tissue origin distribution of myeloma cells, it was found that all four myeloma cell subpopulations were mostly derived from MM, with less distribution of SMMh, and there was no significant difference in the distribution of the three cell cycle phases, which were distributed in all phases (**Figures 3E, F**). This suggested that all four myeloma cell subsets are likely to be malignant cells. **Figures 3G, H** demonstrated the nCount RNA and nFeature RNA scores of all myeloma cells, the C1 *IGHA1+* Myeloma cells of the both nCount RNA and nFeature RNA were low. It was noteworthy that C0 *IGLC3+* Myeloma cells and C3 *IGHG4+* Myeloma cells had higher nFeature RNA scores, indicating that they had active intracellular proliferation, which might play an important role in the growth and metastasis of MM tumors. The expression of TOP5 differential genes in the four myeloma cell subpopulations was shown in **Figure 3I**. The TOP5 differential genes in C0 *IGLC3+* Myeloma cells were *IF127, IGLL5, GSTP1, HLA-B*, and *HLA-C.* The TOP differential genes in C3 *IGHG4+* Myeloma cells were *HBB, HBA2, HBA1*, and *JUNB*. The expression patterns of the myeloma cell subpopulation marker genes in **Figures 3J, K** could be observed that the expression of IGLC3 and IGHA1 was higher in C0 *IGLC3+* Myeloma cells, C1 *IGHA1+* Myeloma cells, and that the expression of *IGHG1* and *IGHG4* was higher in C2 *IGHG1+* Myeloma cells, C3 *IGHG4+* Myeloma cells. The enhanced genes in C0 *IGLC3+* and the diminished genes in the four myeloma cell subpopulations were depicted in volcano plots. The genes that were down-regulated were *IGKC, IGHA1, IGHG4, IGHG3*, and *IGHG1*, and the myeloma cells were *HLA-C, HLA-B, B2M, GSTP1*, and *IGLL5* (**Figure 3L**).

In order to understand the biological functions as well as molecular characteristics of different myeloma cell subpopulations, we performed GOBP enrichment analysis, and the word cloud maps showed that C0 *IGLC3+* Myeloma cells were enriched in the pathways related to reticulum, endoplasmic, targeting, and viral, and C1 *IGHA1+* Myeloma C1 *IGHA1+* Myeloma cells were enriched in electron, triphosphate, purine, atp and other related pathways, C2 *IGHG1+* Myeloma cells were enriched in subunit, ribosomal, burst and other related pathways, C3 *IGHG4+* Myeloma cells were enriched in oxide, nitric, cysteinetype, endopeptidase, electron and other related pathways (**Figure 3M**). Lastly, the enrichment analysis’s findings were further illustrated: C0 *IGLC3+* ATP synthesis and electron transport, the mitochondrial ATP generation and electron transport, respiratory and aerobic electron transport chains, oxidative phosphorylation, and aerobic respiration pathways were all shown to be more abundant in myeloma cells. C1 *IGHA1+* aerobic respiration, respiratory electron transport chain, ATP synthesis linked electron transport, mitochondrial ATP synthesis coupled electron transport, oxidative phosphorylation, and other pathways were enriched in C2 *IGHG1+* in myeloma cells. Aerobic breathing, pulmonary electron transport chain routes, myeloma cells, and electron transport. C2 *IGHG1+* Myeloma cells were enriched in cytoplasmic translation, ribosome biogenesis, ribonucleoprotein complex biogenesis, ribosomal small subunit biogenesis, rRNA processing, and ribosomal large subunit biogenesis. C3 *IGHG4+* myeloma cells were enriched in cytoplasmic translation, ribosome biogenesis, ribonucleoprotein complex biogenesis, ribosomal small subunit biogenesis, rRNA processing, ribosome assembly, and other pathways (**Figure 3N**).Taken together, it suggests that cellular proliferation is more active in C0 *IGLC3+* Myeloma cells and was associated with oxidative phosphorylation, which might regulate metabolic activities through a specific pathway and therefore had a significant impact on the growth and metastasis of MM tumors.

### Proposed time-series analysis of myeloma cell subpopulations

**Figure 4:**
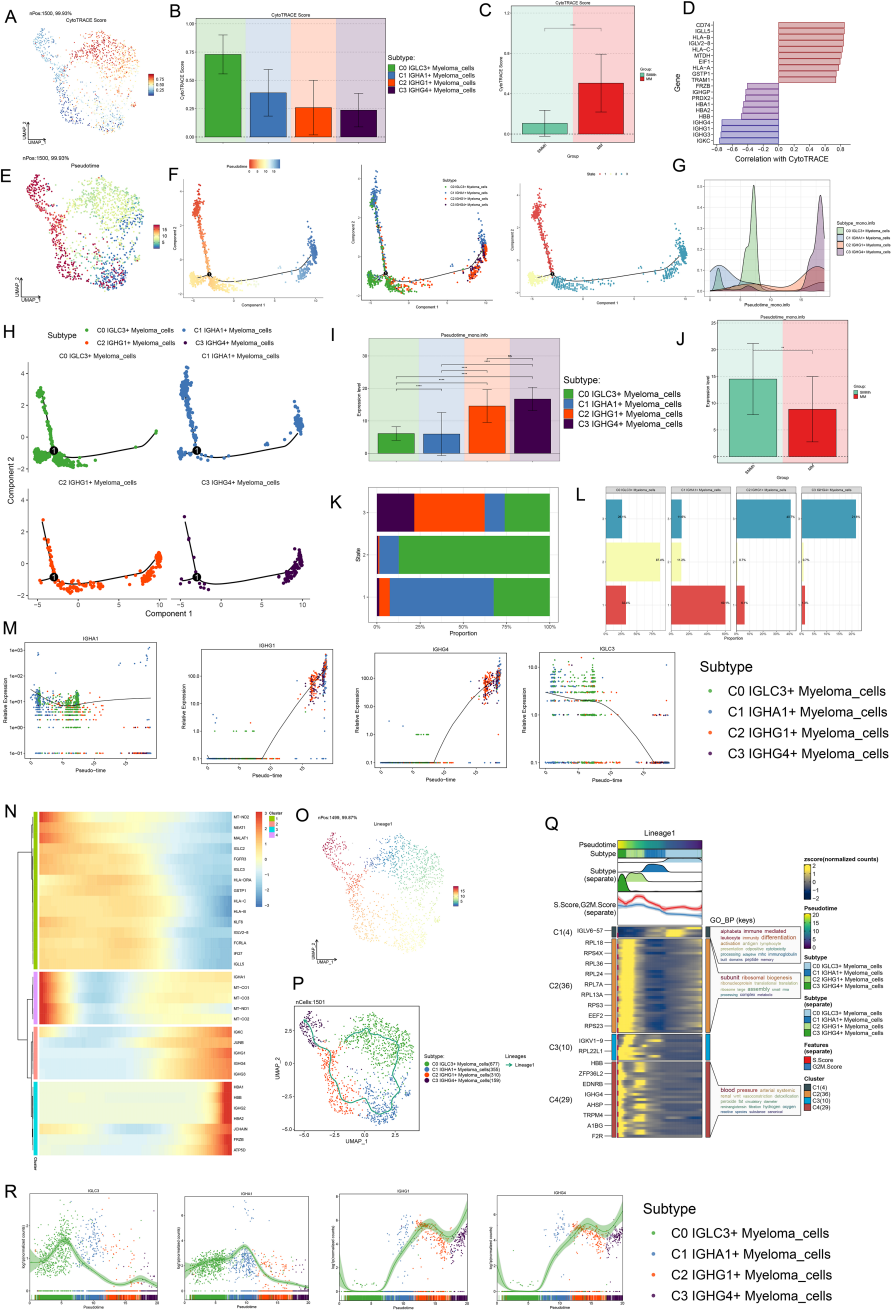
Proposed time-series analysis of myeloma cell subpopulations. **(A, B)** The UMAP and Bar plots showed the results of the stemness ability of four subtypes of myeloma cells calculated based on CytoTRACE. **(C)** Bar plot showed the different levels of differentiation in SMMh and MM. **(D)** Correlation of stemness-related genes in with CytoTRACE. **(E)** UMAP plot demonstrated the order of cell differentiation inferred by Monocle2. **(F)** Two-dimensional trajectory plots demonstrated the differentiation trajectories of myeloma cells labeled with putative chronological order, cellular subpopulations, and putative temporal stages, respectively. **(G)** Ridge plot demonstrated the difference in distribution of the 4 myeloma cell subpopulations across the proposed temporal trajectory. **(H)** 2D trajectory faceted plots demonstrated the distribution of each of the 4 myeloma cell subpopulations across the pseudotime trajectories. **(I, J)** Bar plots demonstrated the differences in the proposed temporal order of the 4 myeloma cell subpopulations as well as different tissue types. **(K, L)** Stacked bar plots demonstrated the distribution of the 4 myeloma cell subpopulations as a percentage of the distribution in the three proposed temporal order trajectory phases. **(M)** Scatter plots demonstrated the distribution of *IGLC3, IGHA1, IGHG1, IGHG4* along with Monocle2 simulated mimetic timing trajectories in different myeloma cell subpopulations. **(N)** Heatmap demonstrated the expression of differential genes along with the pseudotime trajectories. **(O, P)** UMAP plots depicted the temporal dynamics of cell differentiation profiles of four myeloma cell subtypes: C0-C1-C2-C3. **(Q)** Heatmap showed GO-BP pathway enrichment during myeloma cell differentiation. **(R)** Scatter plots showed the distribution of *IGLC3, IGHA1, IGHG1, IGHG4* along with Slingshot-simulated pseudotime trajectories in different myeloma cell subpopulations.

A series of trajectory prediction techniques were used to infer the differentiation process and development trajectory of myeloma cell subsets, to elucidate the differentiation and development relationship among various myeloma cell subsets, and to evaluate the proliferation and differentiation capacity. First, CytoTRACE was used to analyze the cell differentiation and proliferation potential. The results showed that C0 *IGLC3+* myeloma cells had the highest differentiation potential, indicating that C0 *IGLC3+* myeloma cells had the strongest proliferation ability and were naive tumor cells, followed by C1 *IGHA1+* myeloma cells, C2 *IGHG1*+ myeloma cells, and C3 *IGHG4*+ myeloma cells (**Figures 4A, B**). Then, CytoTRACE was used to score cells from different tissues, and it was found that the stemness level of cells from MM was significantly higher than that from SMMh (**Figure 4C**). In addition, it was found that genes such as *IGHG4, IGHG1, IGHG3* and *IGKC* were negatively correlated with CytoTRACE, while genes such as *CD74, IGLL5, HLV2-B* and *IGLV2–8* were positively correlated with CytoTRACE (**Figure 4D**).

Next, we analyzed the subpopulation of myeloma cells by Monocle2, and inferred the cell differentiation trajectory. The results showed that there was a branch point in predicting the development trajectory of myeloma cells, and the whole development trajectory was divided into three states (**Figures 4E, F**). In order to know more clearly the distribution and changes of each myeloma cell subpopulation in the whole development track, we used ridge map and facet maps for visualization (**Figures 4G, H**). The results showed that C0 *IGLC3+* Myeloma cells are mainly distributed in the initial section of the simulated trajectory, namely State1 and State 2. C1 *IGHA1+* Myeloma cells were mainly distributed in the middle stage, namely State 2; C2 *IGHG1+* Myeloma cells and C3 *IGHG4+* Myeloma cells were mainly distributed in the final stage of the trajectory, that is, State3. Further combination **Figure 4I**, the cell differentiation trajectory could be inferred as C0 *IGLC3+* Myeloma cells→C1 *IGHA1+* Myeloma cells→C2 *IGHG1+* Myeloma cells→C3 *IGHG4+* Myeloma cells. By analyzing the differentiation characteristics of different tissue features, it was found that the degree of differentiation of MM was significantly lower than that of SMMh (**Figure 4J**). The cells derived from MM were confirmed to be malignant tumor cells. The percentages of the four myeloma cell subpopulations in the three States (**Figures 4K, L**), C0 *IGLC3+* Myeloma cells accounted for 32.4% in State1 and 87.4% in State2; C1 *IGHA1+* Myeloma cells accounted for 60.1% in State1 and 11.2% in State3; 40.7% of cells in State3 originated from C2 *IGHG1+* Myeloma cells, and 21.6% of cells originated from C3 *IGHG4+* Myeloma cells; in addition, the number of cells in State2 was significantly higher than that in State1, which indicated that C0 *IGLC3+* Myeloma cells and C1 *IGHA1+* Myeloma cells were at the anterior end of the developmental trajectory and are less differentiated, while C2 *IGHG1+* Myeloma cells, and C3 *IGHG4+* Myeloma cells were at the terminal end and were more differentiated. Analyzing the expression changes of markers in the four myeloma cell subpopulations with the predicted developmental trajectories (**Figure 4M**), IGLC3 was the most highly expressed in the initiation segment and gradually decreased with the proposed chronological trajectory, *IGHA1* was relatively evenly expressed throughout the developmental trajectory, and *IGHG1* and *IGHG4* were elevated with the proposed chronological trajectory, and were more highly expressed in the terminal segment. Changes in other genes with the proposed temporal trajectory were shown in **Figure 4N**.

Finally, we performed developmental analysis again using Slingshot and hypothesized 1 cell lineage developmental trajectory, i.e. Lineage: C0 *IGLC3+* Myeloma cells→C1 *IGHA1+* Myeloma cells→C2 *IGHG1+* Myeloma cells→C3 *IGHG4+* Myeloma cells (arrows indicate the direction of cell developmental trajectories) (**Figures 4O, P**). Based on the cell lineage developmental characteristics, GO BP enrichment analysis was performed, and C0 *IGLC3+* Myeloma cells were enriched in alphabeta immune mediated leukocyte, immunity differentiation activation and antigen lymphocyte pathways, C3 *IGHG4+* Myeloma cells were enriched in subunit ribosomal, biogenesis, riponucleoprotein and translational translaton pathways, and C2 *IGHG1+* Myeloma cells were enriched in blood pressure, arterial systemic and reninangiotensin and other related pathways (**Figure 4Q**). The changes in gene expression with the development of the proposed temporal trajectory showed that IGLC3 had the highest expression at the beginning, IGHA1 had the highest expression at the intermediate stage, and IGHG1 and IGHG4z had the highest expression at the terminal segment (**Figure 4R**). In summary, we determined that C0 *IGLC3+* Myeloma cells are malignant tumor cells with high proliferation capacity and low differentiation level, which are important for MM.

### TF regulation and subpopulation-specific activity in myeloma cells

**Figure 5:**
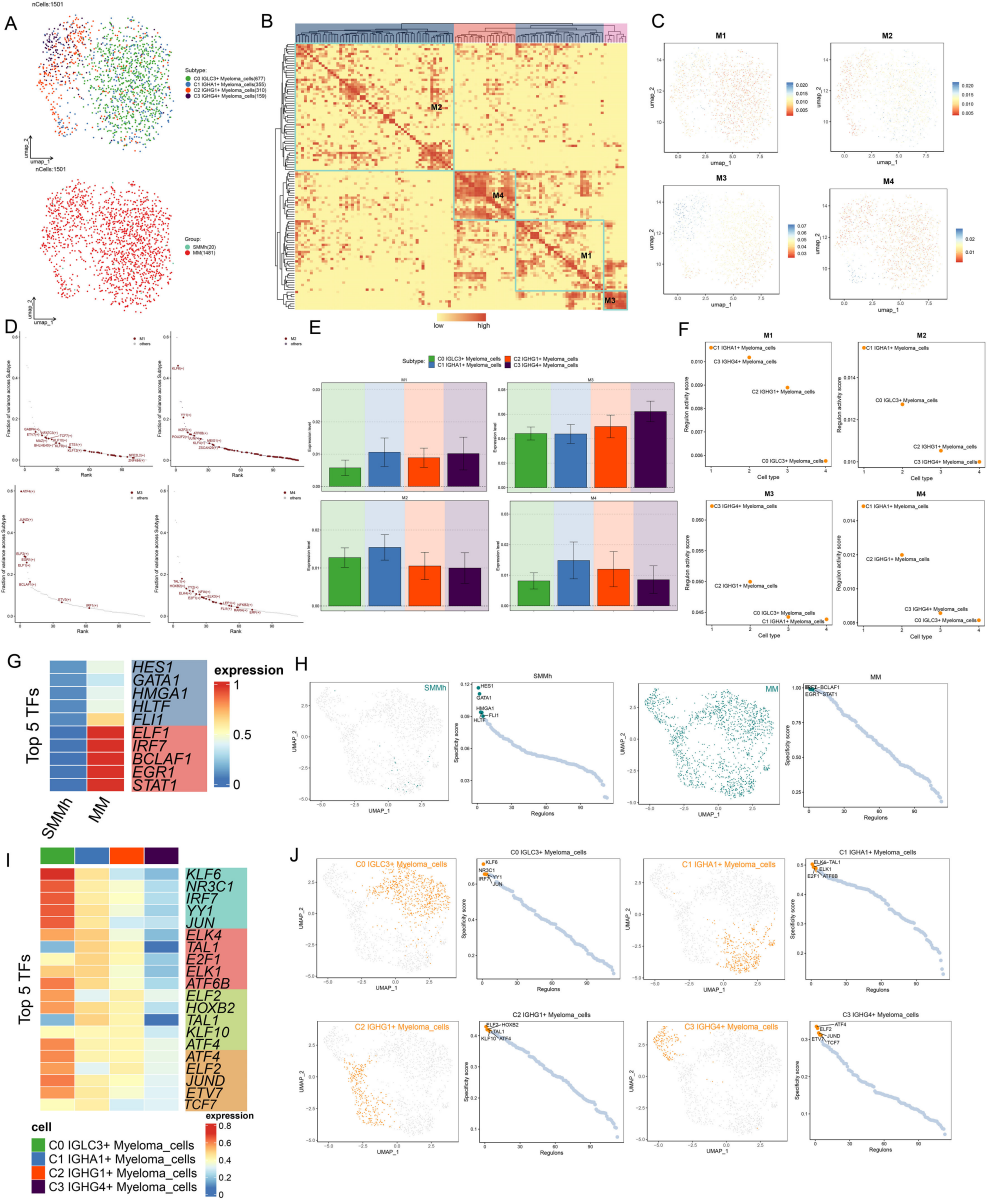
Analysis of TF regulatory activity in myeloma cells. **(A)** UMAP plots showed the distribution of cell subpopulations as well as tissue origin after reclustering analysis based on regulatory activity of myeloma cell TFs. **(B)** Heatmap demonstrated the categorization of endothelial cell TFs into four regulatory modules (M1, M2, M3, and M4) based on the CSl matrix. **(C)** UMAP plots demonstrated the distribution of TFs in the four regulatory modules. **(D)** The TFs in the four regulatory modules were ranked according to fraction of variance across subtype, and their rankings are shown separately. **(E, F)** Bar plots and scatter plots showed the expression levels of TFs and Regulon activity score based on the four myeloma cell subpopulations in the four regulatory modules, respectively. **(G)** Heatmap showed the expression of TOP5 TFs in SMMh as well as MM. **(H)** UMAP plots and scatter plots showed the distribution of TFs in SMMh and MM and Specificity score of Regulon. **(I)** Heatmap demonstrated the expression of TOP5 TFs in four myeloma cell subpopulations. **(J)** UMAP plots and scatter plots demonstrated the distribution of TFs in four myeloma cell subpopulations and specificity score of Regulon.

A protein known as a TF attached to particular DNA sequences, either by itself or in combination with other proteins. It then either promoted or inhibited the recruitment of particular genes to RNA polymerase, controlled gene expression, and influenced the biological processes of cells. Initially, we reclassified myeloma cells according to TFs activity regulation (**Figure 5A**). The UMAP plots of the downward clustering based on the regulatory activities of TFs were less discrete, and the distributions of C0 *IGLC3+* Myeloma cells and C2 *IGHG1+* Myeloma cells were more clearly delineated; similarly, the histological characteristics of four myeloma cell subsets were more favorable to MM. Next, TFs of myeloma cells into four regulatory modules (M1, M2, M3, and M4) (**Figure 5B**), with higher TF regulatory activities in M3 and M4. The distribution of TFs in the four regulatory modules was shown in **Figures 5C, D**. The regulatory activities of GABPA, ETV7, NFATC3 and MAZ were higher in M1, KLF6, YY1, IKZF2 and POU2F2 in M2, ATF4, JUND, ELF2 and EGR1 in M3, and the levels of TAL1, HOXB2, YY2 and ELK4 in M4. The regulatory activities of the four myeloma cell subpopulations varied in different regulatory modules (**Figures 5E, F**), with some degree of regulatory activity of C0 *IGLC3+* Myeloma cells in the M1 and M4 regulatory modules, C0 *IGLC3+* Myeloma cells and C1 *IGHA1+* Myeloma cells in the M2 regulatory module. C2 *IGHG1+* Myeloma cells and C3 *IGHG4+* Myeloma cells in M3 regulatory module had relatively high regulatory activity. Next, we demonstrated the expression of TOP5 TFs in different tissue types as well as in different myeloma cell subpopulations by different visualizations, the top five TFs with the highest regulatory activity in MM were ELF1, IRF7, BCLAF1, EGR1 and STAT1, and the TOP5 TFs in SMMh were HES1, GATA1, HMGA1, HLTF and FLI1 (**Figures 5G, H**); the top five regulators of regulatory activity in C0 *IGLC3+* Myeloma cells were KLF6, NR3C1, IRF7, YY1 and JUN, and the TOP5 TFs in C1 *IGHA1+* Myeloma cells were ELK4, TAL1, E2F1, ELK1 and ATF6B, TOP5 TFs in C2 *IGHG1+* Myeloma cells were ELF2, HOXB2, TAL1, KLF10 and ATF4, TOP5 TFs in C3 *IGHG4+* Myeloma cells were ATF4, ELF2, JUND, ETV7 and TCF7 (**Figures 5I, J**).

### Analysis of TFs and metabolic characteristics of C0 *IGLC3+* myeloma cells

**Figure 6:**
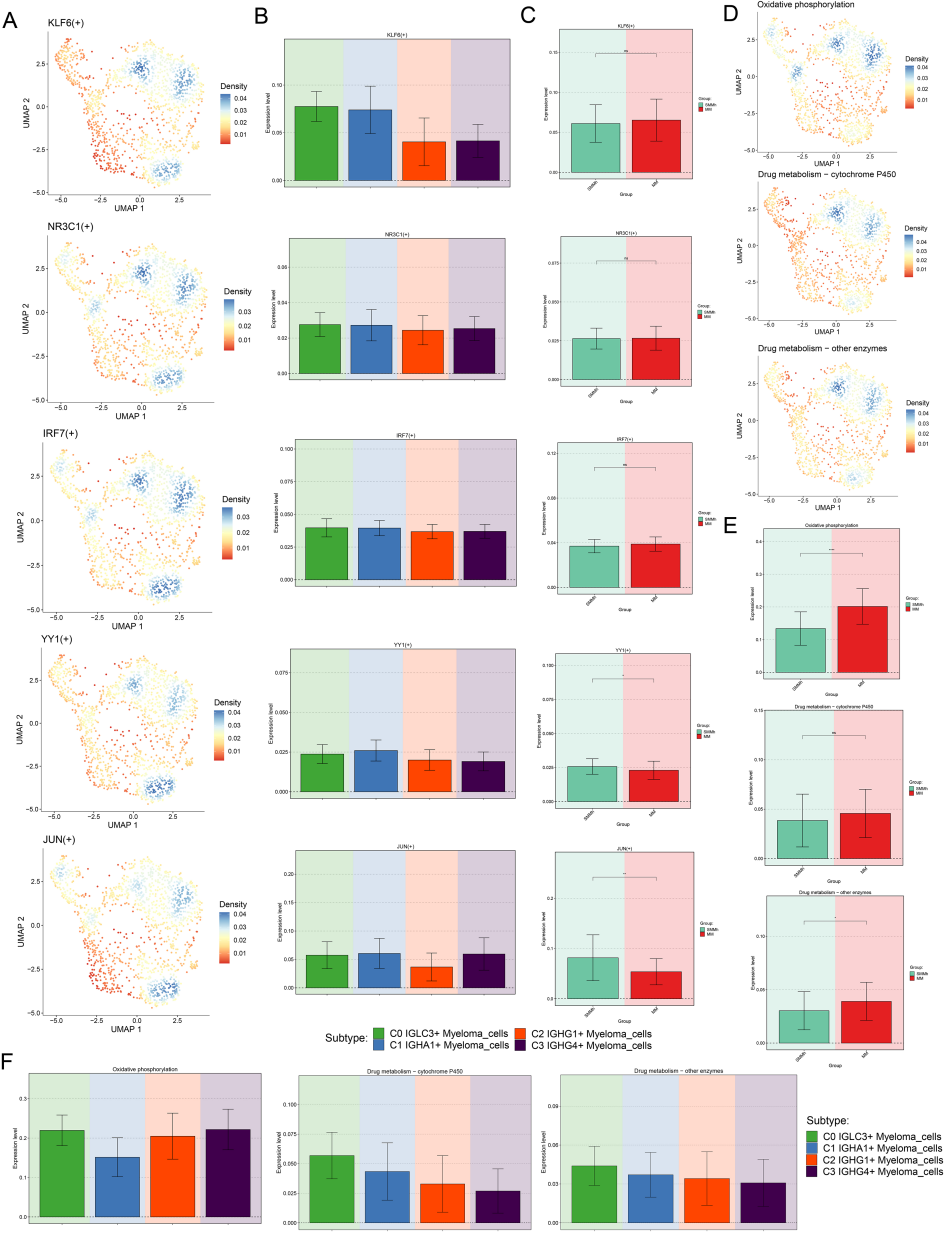
Visualization of TOP5 TFs of C0 *IGLC3+* Myeloma Cells. **(A)** UMAP plots demonstrated the distribution density of TOP5 TFs (KLF6, NR3C1, IRF7, YY1, JUN) of C0 *IGLC3+* Myeloma Cells. **(B)** Bar plots demonstrated the expression levels of KLF6(+), NR3C1(+), IRF7(+), YY1(+), JUN (+) in four myeloma cell subpopulations. **(C)** Bar plots showed the expression levels of KLF6(+), NR3C1(+), IRF7(+), YY1(+), JUN (+) in SMMh as well as MM. **(D)** Levels of regulatory activity of myeloma cell subpopulations under different metabolic pathways. (Oxidative phosphorylation, Drug metabolism-cytochrome P450 and Drug metabolism-other enzymes) **(E)** Differential expression levels of different tissue types under different metabolic pathways. **(F)** Bar plots further demonstrated the regulatory activity of myeloma cell subpopulations under different metabolic pathways.

Next, we further analyzed the regulatory activity of TOP5 TF of C0 *IGLC3+* Myeloma cells, and we visualized the expression of KLF6, NR3C1, IRF7, YY1 and JUN in all cells and different tissue sources (**Figures 6A–C**). The results showed that KLF6, NR3C1, IRF7 and YY1 were all actively regulated in C0 *IGLC3+* Myeloma Cells, C1 *IGHA1+* Myeloma Cells, and JUN was actively regulated in C0 *IGLC3+*Myeloma Cells, C1 *IGHA1+* Myeloma Cells and C3 *IGHG4+* Myeloma Cells. KLF6 was active in MM, YY1 and JUN are active in SMMh, and IRF7 and NR3C1 may be active in MM.

Single cell metabolism analysis was of great significance in revealing cell heterogeneity, disease mechanism and personalized treatment. Finally, we analyzed the metabolic characteristics of myeloma cell subsets, and found that the expression level of Oxidative phosphorylation was higher in C0 *IGLC3+* Myeloma cells and C3 *IGHG4+* Myeloma cells, while the expression levels of Drug metabolism-cytochrome P450 and Drug metabolism-other enzymes were more active in C0 *IGLC3+* Myeloma cells. In addition, all three metabolic pathways were actively expressed in MM (**Figures 6D–F**). The above results indicated that C0 *IGLC3+* Myeloma cells was a subpopulation of MM cells with active cell metabolism, which was related to various metabolic activities.

### Analysis of the interaction between various cell types in myeloma

**Figure 7:**
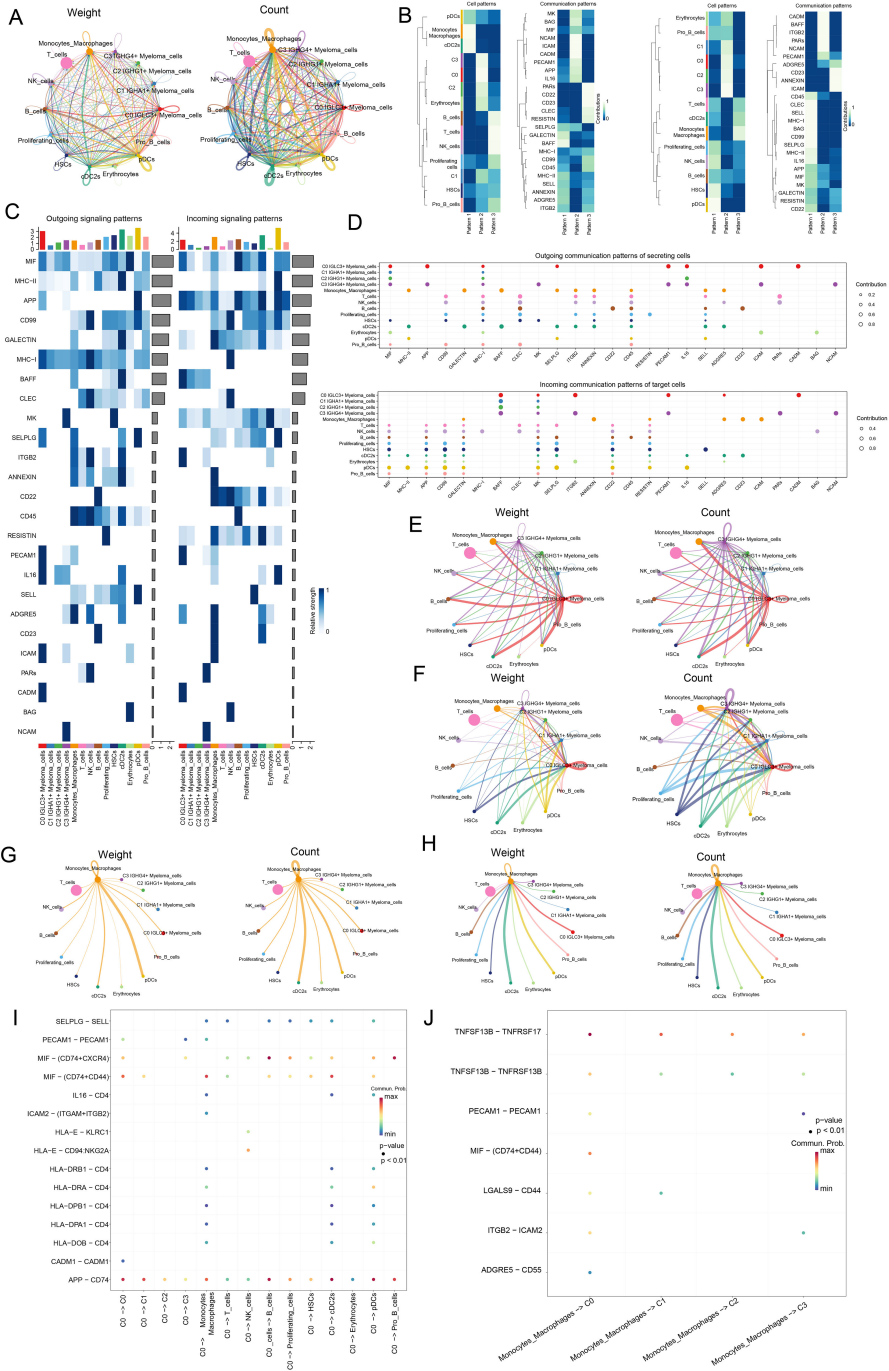
Analysis of the Interaction between Various Cell Types in Myeloma. **(A)** Circle plots showed the weight and quantity of receptor ligand pair interactions between four myeloma cell subsets and all other cells (Monocytes and Macrophages, T cells, NK cells, B cells, Proliferating cells, HSCs, cDC2s, Erythrocytes, pDCs, Pro B cells). **(B)** Heatmaps showed the communication Pattern of outgoing signal (left) and incoming signal (right) between various cells in myeloma. **(C)** Heatmaps showed the communication probability of each communication in Outgoing signaling patterns and Incoming signaling patterns of all cell types. **(D)** Bubble plots showed Outgoing communication patterns of secreting cells and Incoming communication patterns of target cells. **(E)** Screening of four myeloma cell subpopulations for source, circle plots showed the weight (left) and number (right) of cell-cell interactions contributing. **(F)** Screening of four myeloma cell subpopulations for Target, with weight (left) and number (right) contributions of interactions between cells. **(G)** Screening of Monocytes Macrophages for source, where we used circle plots to show the weight (left) and number (right) contributions of interactions between cells. **(H)** Screening of Monocytes Macrophages for Target, with weight (left) and number (right) contributions of interactions between cells. **(I)** Bubble plot demonstrated the interaction of receptor-ligand pairs between C0 *IGLC3+* Myeloma Cells as source and other cells as target. **(J)** Bubble plot demonstrated Monocytes Macrophages as source and four myeloma cell subpopulations as the role of receptor-ligand pairs between targets.

In this study, the researchers aimed to analyze cellular interactions and gain insights into complex biological processes by examining ligand-receptor communication networks through CellChat analysis. Initially, various cell types, including four subpopulations of myeloma cells, T cells, NK cells, B cells, proliferating cells, monocytes, macrophages, HSCs, cDC2s, erythrocytes, pDCs, and pro B cells, were analyzed for intercellular communication and interactions within the cellular microenvironment. **Figure 7A** showed the amount of receptor-ligand pair connections and the strength of relationships among each type of cell. The number of C0 *IGLC3+* Myeloma cells interacting with other cells was relatively high compared to other myeloma cell subpopulations. The relationships among cells and communication networks were investigated using gene expression pattern evaluation methods that were accessible on CellChat. To further investigate the role of proteins in both incoming and outgoing cell signaling, we used heatmaps to display the communication patterns of target cells (incoming signals) and secreting cells (outgoing signals) (**Figure 7B**). The results revealed three outgoing communication patterns of secreting cells (pattern 1, pattern 2, pattern 3) and three ingoing communication patterns of target cells (pattern 1, pattern 2, pattern 3), respectively. We focused on four myeloma cell subpopulations, specifically, in the efferent communication patterns of secretory cells, all four myeloma cell subpopulations interacted with other cells mainly through Pattern 1, and the main pathways functioning in Pattern 1 were MK, MIF, and NCAM. In addition, C0 *IGLC3+* Myeloma cells, C2 *IGHG1+* Myeloma cells and C3 *IGHG4+* Myeloma cells also played an important role through Pattern 3, and the major pathways that played a role in Pattern 3 are APP, IL16 and so on. In the afferent communication pattern of target cells, all myeloma cell subpopulations function through Pattern 1 and Pattern 3, with major pathways such as PARs, NCAM, etc. Compared with other myeloma cell subpopulations, the C0 *IGLC3+* Myeloma cells had the highest combined probability of communication for each pathway in both Outgoing signaling patterns and Incoming signaling patterns, suggesting that C0 *IGLC3+* Myeloma cells were the most strongly interacting myeloma cell subpopulation (**Figures 7C, D**). Notably C0 *IGLC3+* Myeloma cells cross talked with each other through MIF and APP in both Outgoing signaling patterns and Incoming signaling patterns. Next, we screened four myeloma cell subpopulations (**Figures 7E, F**) as well as Monocytes Macrophages (**Figures 7G, H**) for mutual crosstalk patterns when source or target, respectively. Consistent with these results, we found that C0 *IGLC3+* Myeloma cells were the more active myeloma cell subpopulation, which was reflected in the weight as well as the number of cell-cell interactions, and found that the interaction between C0 *IGLC3+* Myeloma cells and Monocytes Macrophages was also stronger. Finally, we investigated the strength of specific receptor-ligand pairs under these pathways and found that C0 *IGLC3+* Myeloma Cells were the source and interacted with other cells mainly through APP-CD74, MIF-(CD74+CXCR4), and MIF-(CD74+CD44) (**Figure 7I**). Similarly, Monocytes Macrophages acted through MIF-(CD74+CD44) with C0 *IGLC3+* Myeloma cells (**Figure 7J**). Taken together, this suggested that APP and MIF were important pathways for myeloma cells to function. Finally, we also analyzed the interactions between Proliferating cells and the four myeloma cell subpopulations, and found that Proliferating cells interacted strongly with C0 *IGLC3+* Myeloma Cells, in which the MIF-(CD74+CD44) protein plays an important role (**Supplementary Figures 1A-C**).

### Cellular crosstalk patterns in the MIF signaling pathway and APP signaling pathway

**Figure 8:**
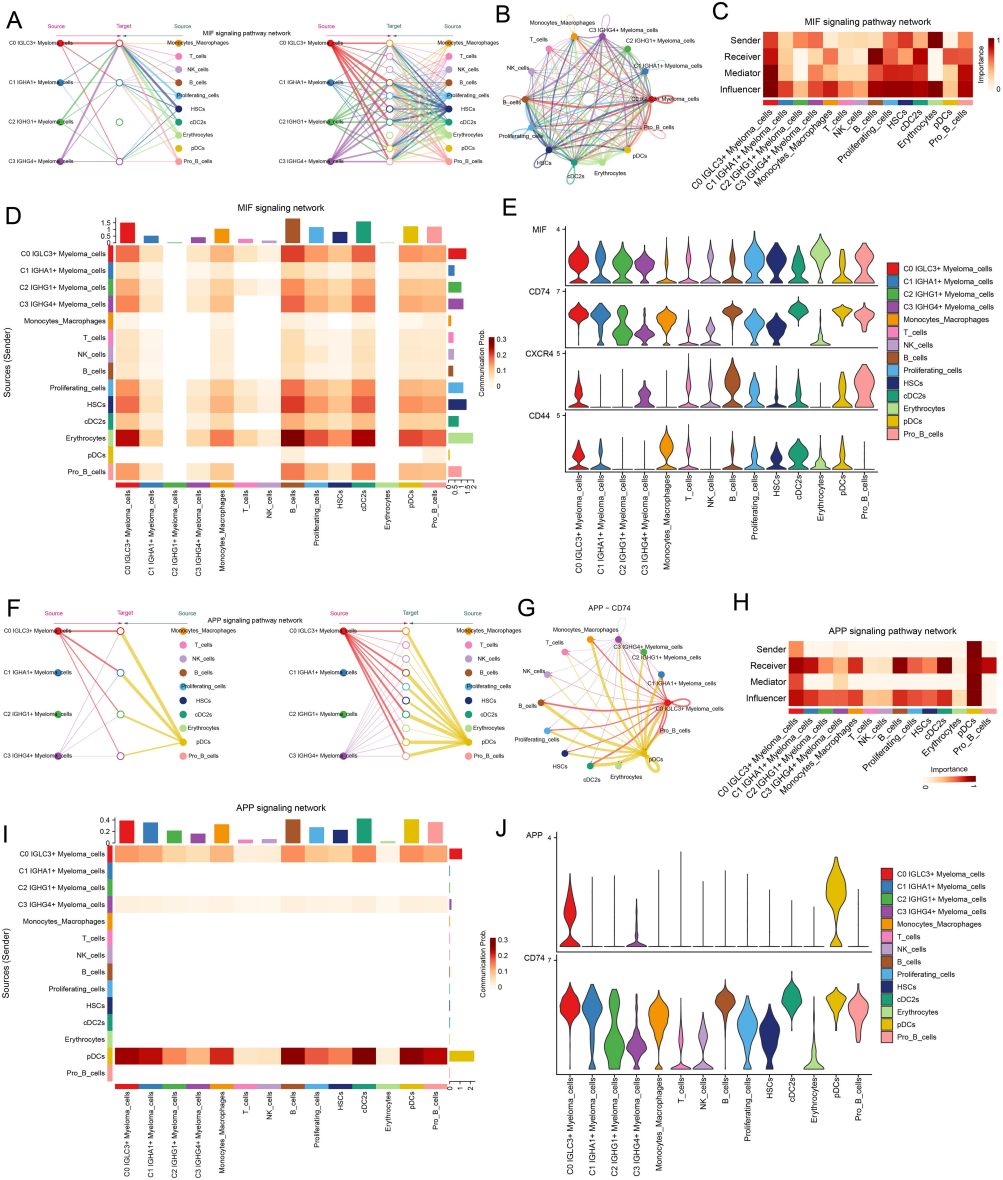
C0 *IGLC3+* Myeloma Cells played important roles through APP signaling pathway and MIF signaling pathway. **(A, B)** Hierarchical plot and circled plot demonstrated autocrine and paracrine interactions of the four myeloma cell clusters with other cells in the MIF signaling pathway. **(C)** Heatmap demonstrated the network centrality scores of different cell clusters in the MIF signaling pathway. **(D)** Heatmap demonstrated the communication probability of different cell clusters in the MIF signaling communication network. **(E)** Violin plot demonstrated the proteins involved in cell-to-cell interactions in the MIF signaling pathway network. **(F)** Hierarchical plot demonstrated cell-to-cell interaction patterns in the APP signaling pathway network. **(G)** Circle plot demonstrated cellular communication patterns of interactions through APP-CD74 protein pairs. **(H)** Heatmap demonstrated network centrality scores of different cell clusters in the APP signaling pathway. **(I)** Heatmap demonstrated the communication probability of different cells under the APP signaling pathway network. **(J)** Violin plot visualized the interacting proteins in the APP signaling pathway.

After revealing the signaling pathways of each cellular interaction, next, we conducted a study on the signaling pathways in the C0 *IGLC3+* Myeloma Cell sand other cells. First, the study was carried out based on the MIF signaling network, and the results showed that all four myeloma cell subpopulations were more active (**Figures 8A, B**). The results of network centrality scoring showed that C0 *IGLC3+* Myeloma Cells played the roles of Sender, Receiver, Mediator, and Influencer roles, with the main role as Influencer (**Figure 8C**). Interaction possibilities between different cell types in response to MIF were depicted in the heatmap (**Figure 8D**). Furthermore, MIF and CD74 proteins were more active in the MIF signaling pathway, according to the violin plot (**Figure 8E**). We next looked at the APP signaling network, and the findings indicated that monocyte/macrophages, C3 *IGHG4+* myeloma cells, and C0 *IGLC3+* myeloma cells had substantial relationships with one another. APP-CD74 was the primary protein pair implicated in this network (**Figures 8F, G**). In the centrality score, the C0 *IGLC3+* Myeloma Cells mainly played the roles of Receiver, Influencer and Mediator. C3 *IGHG4+* Myeloma cells mainly playing the role of Influencer, Monocytes Macrophages Play the roles of Receiver and Influencer (**Figure 8H**). **Figure 8I** displayed the interaction likelihood of several cell groups according to the APP signaling pathway. According to the violin plot, the CD99 signaling pathway primarily functions via the CD74 protein (**Figure 8J**).

### Effect of NR3C1 on proliferation, invasion and metastasis of myeloma cells

**Figure 9:**
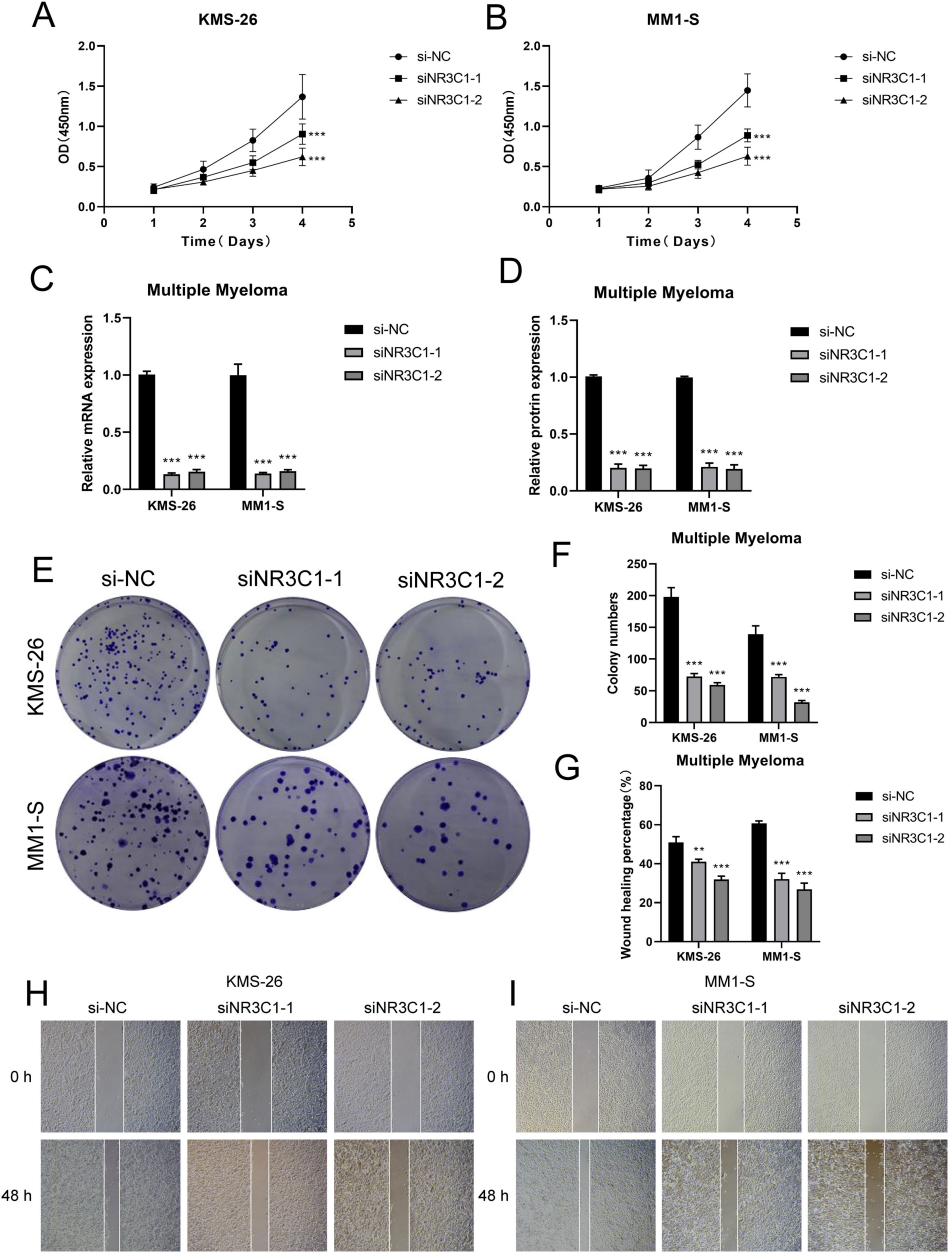
Observe and analyze the changes of cell proliferation ability after NR3C1 knockdown. **(A, B)** CCK-8 was used to detect the changes of cell viability of KMS26 cell line and MM1-S cell line NR3C1 after knockdown. **(C, D)** qRT-PCR was used to detect the mRNA and protein expression levels in KMS26 cell line and MM1-S cell line before and after NR3C1 knockdown. **(E, F)** Colony formation assay was carried out on KMS26 cell line and MM1-S cell line before and after NR3C1 knockdown. **(G–I)** Scratch assay was performed on KMS26 cell line and MM1-S cell line before and after NR3C1 knockdown, **p<0.01, ***p<0.001.

**Figure 10:**
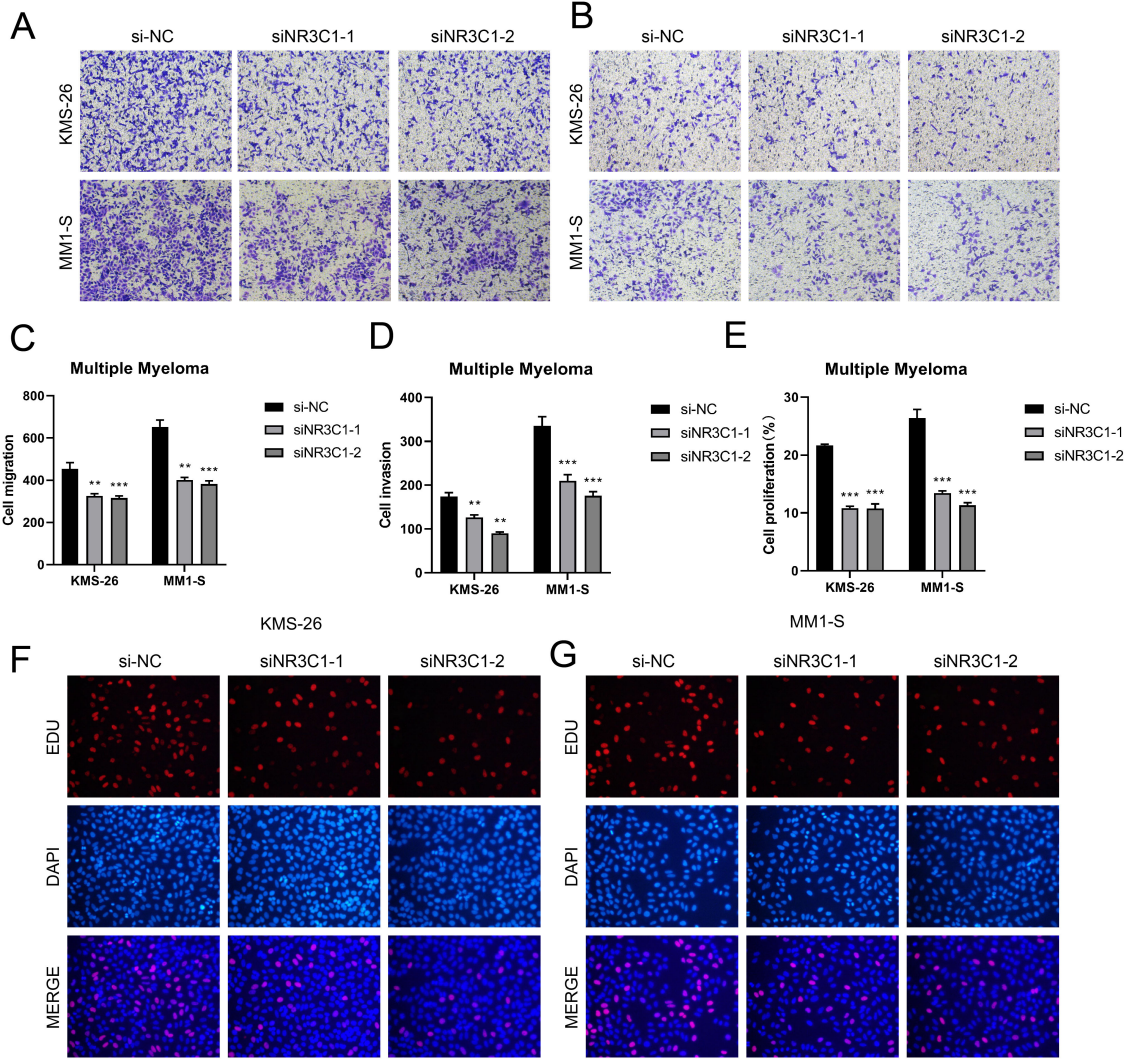
Effect of NR3C1 on proliferation, invasion and metastasis of myeloma cells. **(A–D)** Transwell assay showed that the migration and invasion of KMS26 cell line and MM1-S cell line were significantly reduced after NR3C1 knockdown. **(E–G)** EdU staining showed that the cell proliferation of KMS26 cell line and MM1-S cell line was inhibited after NR3C1 knockdown, **p<0.01, ***p<0.001.

In a previous analysis we found that C0 *IGLC3+* Myeloma cells are naive tumor cell population in MM. Studies had shown that these cells have high proliferation capacity and strong self-renewal properties, which might be key drivers of myeloma development and progression. In C0 *IGLC3+* myeloma cells, NR3C1 exhibited strong regulatory activity, indicating that it might be crucial for controlling cell division and proliferation in this subpopulation. The proliferation and spread of myeloma cells might be facilitated by the elevated expression of NR3C1. NR3C1 not only played an important role in the proliferation and differentiation of myeloma cells, but might also affect tumor growth and progression by regulating cellular metabolic pathways. Therefore, we chose NR3C1 as a target and observed the changes in migration and proliferation ability of myeloma cells by knocking down NR3C1. We chose KMS26 cell line and MM1-S cell line for our experiments, using the method of comparing the negative control and knockdown FOXM1 groups. In the cell viability assay (**Figures 9A, B**), CCK-8 test showed a significant decrease in cell viability after NR3C1 knockdown. The mRNA as well as protein expression levels were found to be significantly reduced after NR3C1 knockdown in myeloma cells using qRT-PCR assay (**Figures 9C, D**). The results of plate cloning assay showed that the colony forming ability of KMS26 cell line and MM1-S cell line was significantly decreased after NR3C1 knockdown (**Figures 9E, F**). According to the findings of cell scratch assay, the two lineages that had NR3C1 broken down had 48-hour scratch widths that were noticeably wider than those of the control group (**Figures 9G–I**). Finally, the results of Transwell assay (**Figures 10A–D**) as well as EdU staining (**Figures 10E–G**) showed that the proliferation and migratory invasion of KMS26 cell line and MM1-S cell line were significantly reduced after NR3C1 knockdown. Through these experiments, we discovered that myeloma cells with NR3C1 knockdown had reduced invasion, migration, and proliferation—all of which were critical for the malignant evolution of myeloma.

## Discussion

Understanding the intricate interactions between tumor cells and the bone marrow (BM) microenvironment remains a major challenge in MM, an incurable BM-resident plasma cell malignancy (1). In this study, we employed scRNA-seq to comprehensively characterize the cellular heterogeneity of high-risk smoldering multiple myeloma (SMMh) and overt MM at an unprecedented resolution. By delineating the complex cellular crosstalk within the BM niche, we identified critical subpopulations and signaling pathways that potentially drive disease progression and facilitate immune evasion in MM.

Importantly, the immune responses within the BM were not only shaped by tumor cell proliferation but were also influenced by the neuroimmune interactions that drive neuroinflammation, which played a crucial role in tumor progression. The BM microenvironment itself was known to be a site of neuroinflammation, where sensory nerve fibers released neurotransmitters and neuropeptides that modulated immune responses, contributing to immune escape mechanisms in MM (70). Such interactions highlighted the complexity of tumor immunology, where neural activity and immune regulation were tightly intertwined. Studies had shown that stress-induced neuroinflammation can impact immune responses in tumors, including MM, by modulating cytokine profiles and immune cell functions, which might influence tumor progression and resistance to therapies (71). This suggested that the nervous system could be an underappreciated but critical player in regulating tumor microenvironments and immune modulation.

We investigated the differentiation trajectories of malignant plasma cells and identified four major myeloma subpopulations: C0 IGLC3+, C1 IGHA1+, C2 IGHG1+, and C3 IGHG4+ myeloma cells. These subclusters displayed distinct molecular signatures, differentiation states, proliferative capacities, and metabolic profiles, reflecting the cellular heterogeneity and dynamic evolution of MM. Among them, the C0 IGLC3+ subset stood out with the highest stemness score and the lowest differentiation level as determined by CytoTRACE, alongside elevated proliferative activity based on cell cycle analysis. These features suggest that C0 cells may represent an initiation-like population at an early stage of differentiation, with the potential to drive MM progression. Moreover, comparative analysis revealed a significant enrichment of C0 IGLC3+ cells in more advanced disease stages (e.g., MM compared to SMM), implicating this subpopulation in the transition from indolent to aggressive disease. In line with prior studies and our own findings, C0 cells may contribute to disease advancement through mechanisms such as immune evasion and interactions with the bone marrow stromal microenvironment. Notably, their transcriptional profile also hinted at potential involvement in neuroimmune regulatory pathways, suggesting a complex interplay between intrinsic tumor properties and extrinsic neural-immune cues.

Our functional analysis revealed that the C0 and C3 subpopulations were enriched for pathways involved in oxidative phosphorylation, a key metabolic process linked to rapid cell proliferation and tumor metastasis (72). This aligns with recent findings in cancer neuroimmunology, which had shown that metabolic pathways regulated by neuropeptides and stress responses could influence tumor aggressiveness (73). In this context, the activation of oxidative phosphorylation could be a mechanism through which MM cells escape immune surveillance, aided by neuroinflammatory signals that promote tumor growth.

Gene expression analysis further supports the hypothesis that these subpopulations contributed to immune evasion. For example, genes like *HLA-C, HLA-B*, and *GSTP1* were highly expressed in the C0 subpopulation, suggesting a role in antigen presentation and immune escape. In contrast, the C3 subpopulation, enriched in genes like *HBB* and *JUNB*, may contribute to oxidative stress regulation and cell cycle progression, suggesting a more mature, functionally active tumor cell. These findings were consistent with the growing understanding that neuroinflammation, often triggered by neural activity, can promote immune evasion by modifying tumor cell behavior and influencing immune checkpoint expression (74).

Our temporal trajectory analysis further refined the differentiation paths of myeloma cells, revealing that the C0 subpopulation lies at the early stage of differentiation, while the C3 subpopulation represents more differentiated, functionally active cells. These insights suggested that the early-stage C0 *IGLC3+* cells, with their high stemness, could be crucial for tumor initiation, while the later-stage C3 cells, enriched for oxidative stress regulation, might play a role in tumor progression and resistance to treatment. This temporal progression mirrors immune evasion strategies, where early-stage myeloma cells might evade immune surveillance by mimicking the effects of neuroinflammatory signaling, while later-stage cells might further complicate immune responses through adaptive immune resistance.

Among other cancers, *IGLC3* was identified as a novel prognostic biomarker for intestinal-type gastric cancer (IGC), and its expression level was closely correlated with the onset and progression of IGC. In addition to being a possible biomarker for intestinal-type gastric cancer, *IGLC3* might be crucial in controlling the immunological milieu and the development of tumors (75); IGLC3 expression was significantly upregulated in cervical cancer (CESC) tissues was significantly up-regulated and associated with multiple clinical and molecular features, which might be involved in tumor regulation by affecting the immune microenvironment and tumor cell properties, and has potential value as a diagnostic and therapeutic target (76); *IGLC3* was one of the important marker genes for B-cells in lung adenocarcinoma (LUAD), which was closely related to B-cell recognition and function, and studies had shown that significant differences in immunoreactivity in different metabolic subtypes and that *IGLC3* might play a linking role between metabolism and immune regulation (77). We hypothesized that C0 *IGLC3+* myeloma cells are closely linked to the progression of MM as a result of these findings. Increased *IGLC3* expression levels and the discovery of mutations in the disease stated clearly imply that *IGLC3* plays a role in the onset and progression of disease.

We analyzed the regulatory activity of TOP5 TFs in C0 *IGLC3+* Myeloma cells and found that KLF6 was actively regulated in MM, and IRF7 and NR3C1 might be active in MM. In addition, IRF7 and NR3C1 might be active in MM. The role of KLF6 in tumors was dichotomous: wild-type KLF6 (wtKLF6) acts as a tumor suppressor gene that inhibited tumor growth; while its splice variant KLF6-SV1 promoted tumor progression, drug resistance, and metastasis (78). Modulation of the expression levels of KLF6 and its variants, especially in hematological tumors such as MM and CLL, provided new potential strategies for precision therapy and overcoming drug resistance (79, 80). NR3C1 encoded the glucocorticoid receptor (GR), a nuclear receptor TF that regulated the expression of multiple genes involved in cell proliferation, apoptosis, metabolism, and inflammatory responses (81). Upregulation of NR3C1 in certain tumors might cause oncogenes to become active or alter the tumor microenvironment to encourage the migration and multiplication of cancer cells. The nuclear factor-κB (NF-κB) signaling pathway controled NR3C1 in breast cancer cells (82). Breast cancer cell migration and proliferation were linked to up-regulation of NR3C1. By drastically lowering NR3C1 expression, NF-κB inhibition slowed the growth of tumors. In previous studies, up-regulation of NR3C1 was associated with enhanced cell proliferation and migration, possibly through metabolic regulation and signaling pathway interactions.NR3C1 and its related regulators (e.g., MDM4, SETBP1, and NF-κB) were potential therapeutic targets, and tumor progression could be inhibited and treatment enhanced by modulating NR3C1 expression (83, 84). IRF7 (interferon regulatory factor 7) was an important TF mainly involved in the up-regulation of type I interferon (IFN), which exerted antiviral immunity and apoptosis-inducing effects. IRF7 regulated gene expression through activation of downstream signaling molecules (e.g., IFNB) in a variety of cell types, which were often associated with apoptosis and proliferation (85). IRF7 could regulate tumor microcirculation through the regulation of NR3C1 expression under some environments could promote tumor cell proliferation by regulating the tumor microenvironment. IRF7 was a significant immune-related gene, and myeloma patients used its expression level as a prognostic indicator to help guide their individualized treatment. Through multi-level control, YY1 influenced drug sensitivity, apoptosis, and proliferation in MM. By stabilizing m6A-modified YY1 mRNA, increasing the transcription of miR-27a-3p, and favorably regulating TUG1, YY1 influenced drug sensitivity, apoptosis, and proliferation in MM (86). The JUN gene encoded the c-Jun protein, a significant TF belonging to the AP-1 family that was essential to the development of cancer, particularly in controlling the proliferation and death of tumor cells. By controlling the expression of several genes linked to the cell cycle and proliferation, c-Jun stimulates the growth of tumor cells. Its activation could lead to increased expression of cell cycle proteins (e.g. Cyclin D1), accelerating cell cycle progression and thus enhancing cell proliferation.

We discovered coordinated contacts between C0 *IGLC3+* myeloma cells and other cell types by using CellChat communication pattern analysis. This method offered important new information on the cellular relationships in the myeloma microenvironment. We were able to uncover important connections between various cell types by delving further into the cellular interactions in MM, which provided insight into the dynamic intercellular communication within the tumor microenvironment.

Our research specifically demonstrated how C0 *IGLC3+* myeloma cells coordinate their interactions with different cell types. Through a network of MIF and APP communication routes, we were able to determine how these cells interact with the surroundings. This complex intercellular communication was essential to the onset and course of MM. Certain communication patterns and the signaling pathways that go along with them were identified by our investigation. Interestingly, we discovered that a subset of C0 *IGLC3+* myeloma cells is associated with the MIF signaling pathway, indicating its significance in the signaling network of this subpopulation. These myeloma cells’ biological activities and functions were probably significantly influenced by the MIF pathway. APP signaling regulates the interaction between myeloma cells and bone marrow stroma through CD74 and CXCR4 receptors, affects the adhesion of myeloma cells to the bone marrow microenvironment, and leads to immune evasion by altering immune cell responses. MIF interacts with CD74 and CXCR4 on myeloma cells to promote their proliferation, survival, and immune evasion, and also supports the inflammatory environment in the bone marrow to promote tumor progression and immune suppression (87). The specific receptor-ligand pairs such as CX3CL1-CX3CR1, NGF-NTRK1, APP-CD74, and MIF-CD74 highlight key signaling axes that influence the communication network in the myeloma microenvironment, suggesting potential therapeutic targets for disrupting these pathways and improving treatment outcomes in multiple myeloma.

Notably, the present study revealed that the C0 and C3 subpopulations have important effects on MM growth and drug resistance through metabolism-related pathways. The notable concentration of pathways like ATP generation and oxidative phosphorylation in particular raised the possibility that these metabolic characteristics could be used as therapeutic targets. The intricate involvement of the bone marrow microenvironment was directly linked to both medication resistance and the progression of MM. The immunological escape mechanism of MM might be significantly impacted by variations in the quantity and activity of immune cell subpopulations in the tumor microenvironment. For example, monocyte-macrophages exhibited higher cell stemness scores and functional activity, suggesting that they might play important pro-inflammatory and pro-tumorigenic roles in supporting tumor growth. This study provided important new insights into the precise typing and personalized treatment of MM through systematic analysis at the single-cell level. We proposed that different myeloma cell subpopulations might influence disease progression and therapeutic response through unique functional pathways and cell-to-cell interaction mechanisms. This finding not only deepened the understanding of MM heterogeneity, but also provided a theoretical basis for the future development of precision therapeutic strategies targeting specific cell subpopulations.

However, this study still had some limitations. First, although we analyzed samples from multiple patients, the sample size was more limited and may not be sufficient to comprehensively cover all subtypes of MM. In addition, the proposed time-series analysis provided a speculative model for the differentiation trajectory of myeloma cells, but further experimental validation was still needed. Future studies should combine *in vitro* and *in vivo* functional experiments to further validate the functions of myeloma cell subpopulations and their potential therapeutic targets.

To sum up, this study demonstrated the variety of tumor cells and how they interact with the microenvironment in MM, which offered important hints for comprehending the disease’s pathogenic mechanisms and creating new treatment alternatives.

## Conclusion

Our research offered fresh perspectives on the cellular variety and developmental paths of MM, highlighting the critical role of neuroimmune interactions and neuroinflammation in shaping the tumor microenvironment. These findings underscored the potential for targeting both tumor cell biology and the neuroimmune axis to improve therapeutic strategies in MM, particularly for overcoming resistance mechanisms. The identification of key subpopulations and their associated pathways offered promising targets for novel, personalized therapies that address the complex interactions between tumor cells, the BM microenvironment, and neuroinflammatory signals, providing a new avenue for MM treatment.

## Data Availability

The original contributions presented in the study are included in the article/**Supplementary Material**. Further inquiries can be directed to the corresponding author/s.
