# Supplementary figures and images for: Decoding multiple myeloma: single-cell insights into tumor heterogeneity, immune dynamics, and disease progression
Source: Front Immunol. 2025 May 8;16:1584350. doi: 10.3389/fimmu.2025.1584350 (PMC12095158; doi:10.3389/fimmu.2025.1584350)

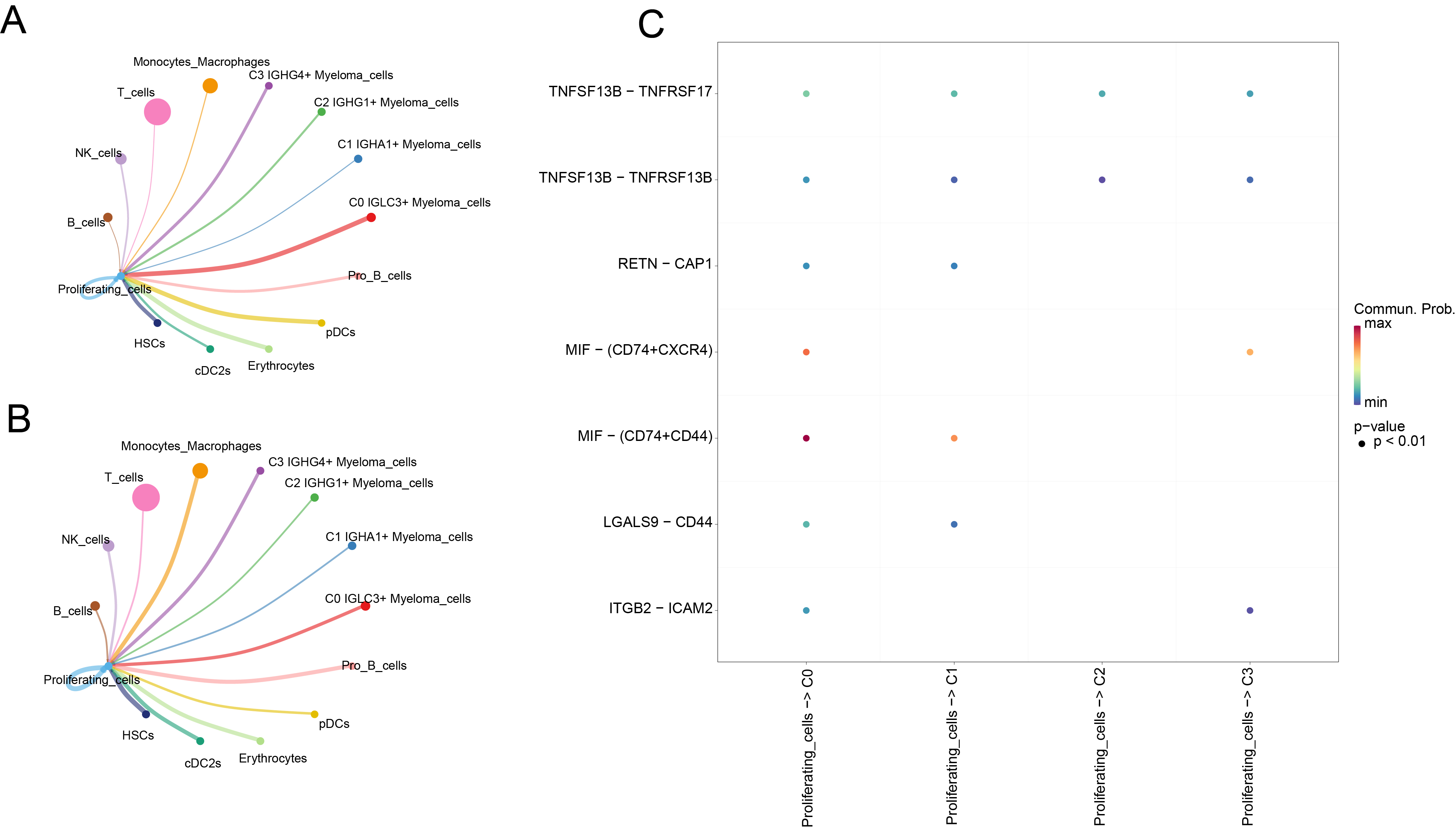

Supplement: Supplementary file 1 [file Image1.jpeg]
